# Stannous colloid mixed with indocyanine green as a tracer for sentinel lymph node navigation surgery

**DOI:** 10.1038/s41598-022-21420-z

**Published:** 2022-10-12

**Authors:** Yiting Zhang, Tomoya Uehara, Taro Toyota, Ryusuke Endo, Hisahiro Matsubara, Hideki Hayashi

**Affiliations:** 1 Department of Frontier Surgery, Graduate School of Medicine, Chiba University, Inohana, Chiba-shi chuo-ku, Chiba, Japan; 2 Department of Molecular Imaging and Radiotherapy, Graduate School of Pharmaceutical Sciences, Chiba University, Inohana, Chiba-shi chuo-ku, Chiba, Japan; 3 Department of Graduate School of Arts and Sciences, The University of Tokyo, Komaba Meguro-ku, Tokyo, Japan; 4 Department of Medical System Engineering, Graduate School of Engineering, Chiba University, Yayoi-cho, Chiba-shi inage-ku, Chiba, Japan; 5 Center for Frontier Medical Engineering, Chiba University, Chiba, Japan

**Keywords:** Preclinical research, Metastasis, Medical imaging

## Abstract

The combined use of a vital dye and radioactive colloid reportedly performs better in detecting sentinel lymph nodes (SLNs) for cancers than the use of either of them alone. However, especially for gastric cancer, two endoscopic procedures are required to administer these two tracers, which burdens the patients and practitioners. Here we propose the use of stannous colloid (SnC) mixed with indocyanine green (ICG) as a new mixed tracer (SnC–ICG); its characteristics were investigated in vivo and in vitro to estimate its usefulness for SLN navigation. The tracers were administered to rats and the accumulation of radioactivity and/or near-infrared fluorescence were evaluated in the regional lymph nodes (LNs) using single positron emission computed tomography and near-infrared fluorescence imaging, respectively. SnC–ICG showed significantly better clearance from the injection site and better migration to primary LNs than the single administration of SnC or ICG aqueous solution. SnC–ICG demonstrated a wide particle size variability, stabilized to 1200-nm upon the addition of albumin in vitro; These properties could contribute to its behavior in vivo. The use of SnC–ICG could contribute better performance to detect SLNs for gastric cancer with less burden on both patients and medical practitioners.

## Introduction

In recent years, sentinel lymph node (SLN) navigation has gained much attention as a tool that can reasonably minimize surgical invasiveness for the treatment of various malignant diseases^1–3^. SLNs are defined as the first one or first few lymph nodes (LNs) which receive lymphatic flow from the primary tumor, and the absence of metastasis in the SLNs indicates no metastasis in the other regional LNs^4^. Therefore, the extent of the lymphatic dissection could reasonably be reduced based on the metastatic status of the nodes revealed intraoperatively. Survival rates after surgeries without regional LN resection in patients without SLN metastasis are comparable with those after conventional radical surgeries^5^. This SLN navigation surgery was first applied to the treatments for skin melanoma^4^ and breast cancer^2^, and subsequently to gynecologic and other solid malignancies^6^. Radioactive colloid or vital dye is used to detect SLN^7,8^. Although radioactive colloids require radiation-controlled handling area and specific visualizing equipment such as a gamma camera or single positron emission computed tomography (SPECT), they show long retention in the LNs, enabling a quantitative evaluation from outside the body with high sensitivity^3,9^. In contrast, vital dyes, such as isosulfan blue, patent blue violet, and indocyanine green (ICG), are easy to handle, inexpensive, and can help visualize every lymphatic route to the SLNs without the need for a detection equipment. The near-infrared (NIR) fluorescence properties of ICG have recently attracted the attention of surgeons as they allow the penetration of biological tissues up to a depth of approximately 1 cm; ICG can be detected with better sensitivity than other dyes using NIR fluorescence video systems^10–12^. However, vital dyes must be administered intraoperatively because they tend to diffuse quickly and reach secondary or downstream LNs almost immediately.

Single use of these tracers has achieved clinically practical identification rates and accuracy levels, while their combined use has shown an improved performance^13–15^. Therefore, a multicenter clinical trial designed to evaluate the usefulness of SLN navigation in gastric cancer surgery in Japan^16^ recommended the combined use of technetium-99m (^99m^Tc)–stannous colloid (SnC) and a vital dye such as ICG or indigocarmine. SLN navigation for gastric cancer surgery could enable function-preserving and less invasive procedures by reducing the required degree of gastric and regional LN resection^17^.

Nevertheless, differences in the pharmacokinetics of the two types of tracers were noted, and a multimodal tracer that could be detected with both NIR and radioactive methods was proposed to overcome this problem^18^. This multimodal tracer was constructed from human serum albumin aggregates and ICG, and it has already been adopted into the clinical management routine of various cancers^19–23^ in several hospitals in European countries. However, this ICG-^99m^Tc-nanocolloid is not available in our country and its utility in gastrointestinal surgeries remains unclear.

In this context, Araki et al.^24^ reported that, in an animal model, the addition of phytate colloid to ICG significantly prolonged the retention of fluorescence in primary LNs and retarded its migration to downstream nodes. However, they did not analyze the mixture’s particle profile; The use of phytate colloid for visceral organs is tricky because it reportedly accumulates heavily in the liver^24^ and behaves differently in rodents and primates^25^. Therefore, we tested the utility of the SnC and ICG mixture (SnC–ICG), both approved by the Pharmaceuticals and Medical Devices Agency in Japan for the detection of SLN.

The combined SnC–ICG administration revealed a significantly better flow from injection sites and retention in primary LNs than the administration of SnC or ICG alone. We also investigated the physical and chemical characteristics of this mixed tracer and the results revealed that the interaction between SnC and ICG contributes to its behavior in vivo.

## Results

### Biodistribution analyses of ICG and SnC–ICG using NIR fluorescence imaging

**Figure 1:**
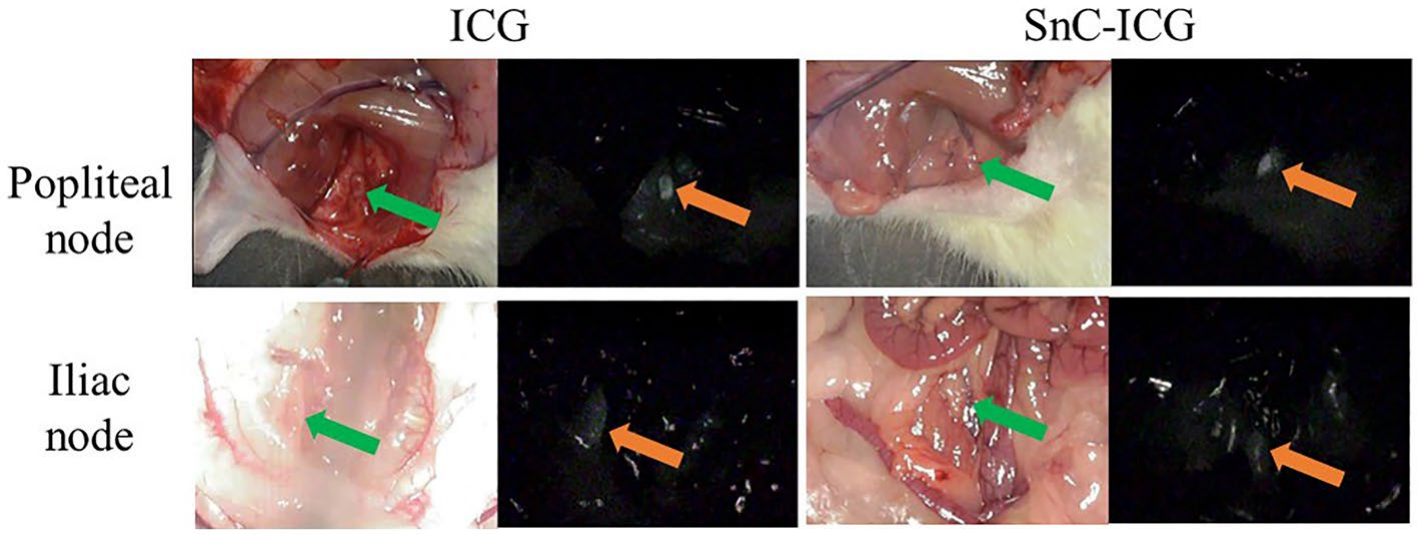
Appearance of regional lymph nodes (LNs) after the administration of indocyanine green aqueous solution (ICG) or a stannous colloid/ICG mixed tracer (SnC–ICG) under bright-field (left) and near-infrared (NIR) fluorescence (right) cameras. The arrows indicate the regional LN. Fluorescent nodes are rather ambiguous in this figure because these images were extracted from movies captured by NIR fluorescence camera with long focal distance for clinical use and high background due to NIR surface-reflections. These nodes were obviously identified with original movies S1 (popliteal node with ICG), S2 (iliac node with ICG), S3 (popliteal node with SnC–ICG), and S4 (iliac node with SnC–ICG).

The NIR fluorescence camera revealed an accumulation of fluorescence in the popliteal LNs of all the evaluated rats at 18 h after the administration of ICG aqueous solution or SnC–ICG (Fig. 1, upper row). The iliac nodes were also examined and generally appeared less fluorescent than the popliteal nodes (Fig. 1, lower row). Fluorescence was noted in the iliac nodes of all rats administered SnC–ICG versus in only three of four rats administered ICG aqueous solution alone (data not shown). The inguinal, caudal, and renal LNs were examined and did not show fluorescence.

**Figure 2:**
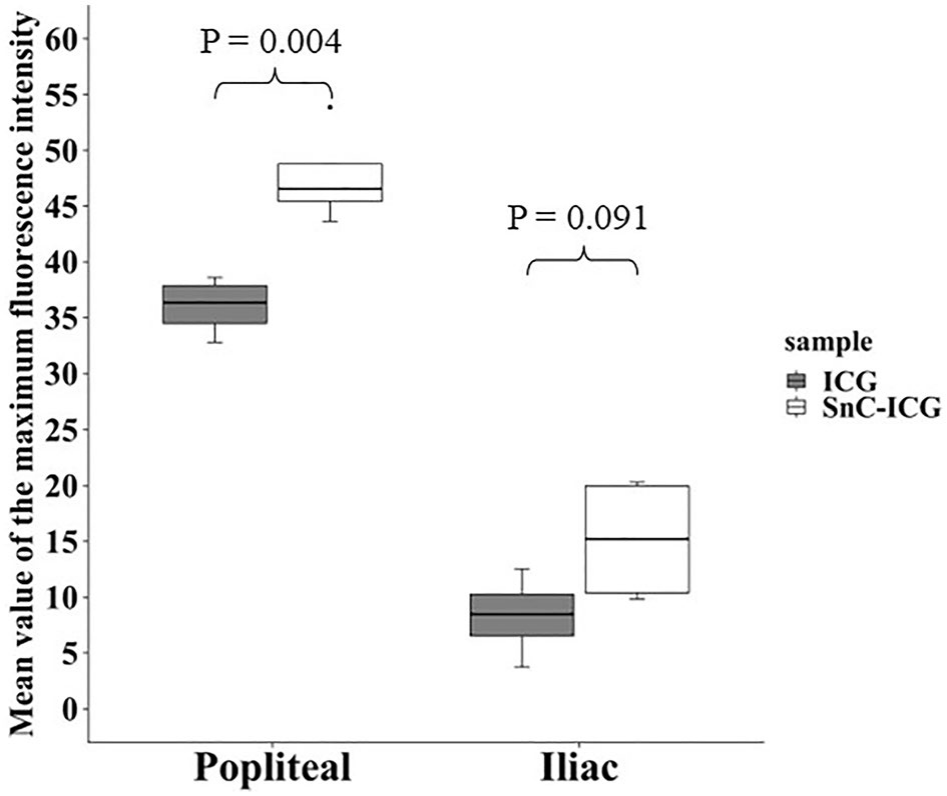
Distribution of maximum fluorescence intensity detected by a near-infrared fluorescence camera in the popliteal or iliac lymph nodes (LNs). ICG, LN from rats administered indocyanine green aqueous solution (n = 4); SnC–ICG, LN from rats administered a stannous colloid/indocyanine green mixed tracer (n = 4). Mean values of the maximum fluorescence intensity of the popliteal or iliac LNs were estimated by ImageJ software.

Distribution analyses of the NIR fluorescence intensities of the popliteal or iliac nodes showed significantly stronger fluorescence in the popliteal nodes (p = 0.004) and relatively stronger fluorescence in the iliac nodes (p = 0.091) of the rats administered with SnC–ICG than those administered ICG aqueous solution alone (Fig. 2).

**Figure 3:**
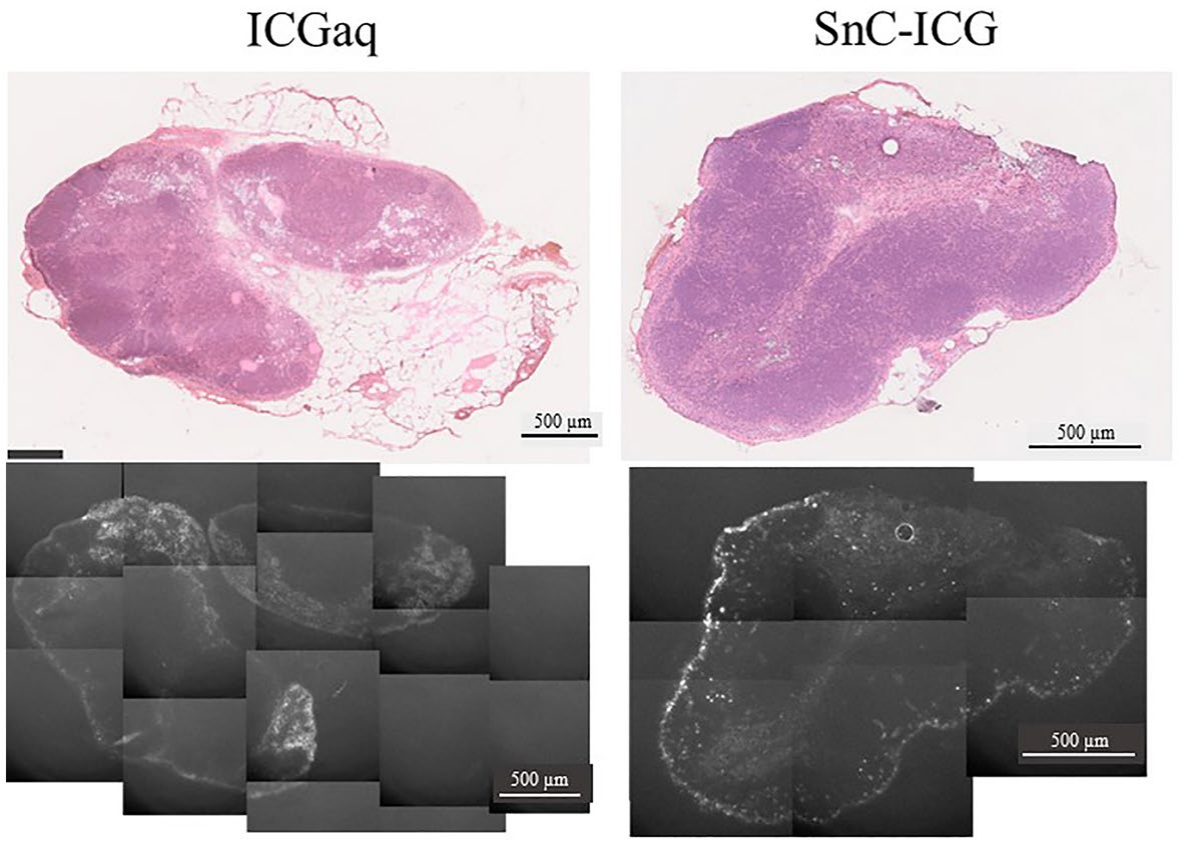
Histological findings of popliteal lymph nodes (LNs) from rats administered indocyanine green aqueous solution (ICGaq) or stannous colloid/indocyanine green mixed tracer (SnC–ICG). Bright-field images of frozen sections stained with hematoxylin–eosin are shown in the upper row, while near-infrared fluorescence images of the unstained section are shown in the lower row.

A histological analysis of the popliteal LN showed disseminated fluorescence in almost all LNs in rats administered ICGaq or SnC–ICG; however, the fluorescence in the marginal sinuses was stronger in the rats administered SnC–ICG versus ICG aqueous solution (Fig. 3).

### Biodistribution analyses of ^99m^Tc-SnC and ^99m^Tc-SnC–ICG using NIR fluorescence camera and SPECT/CT imaging

**Figure 4:**
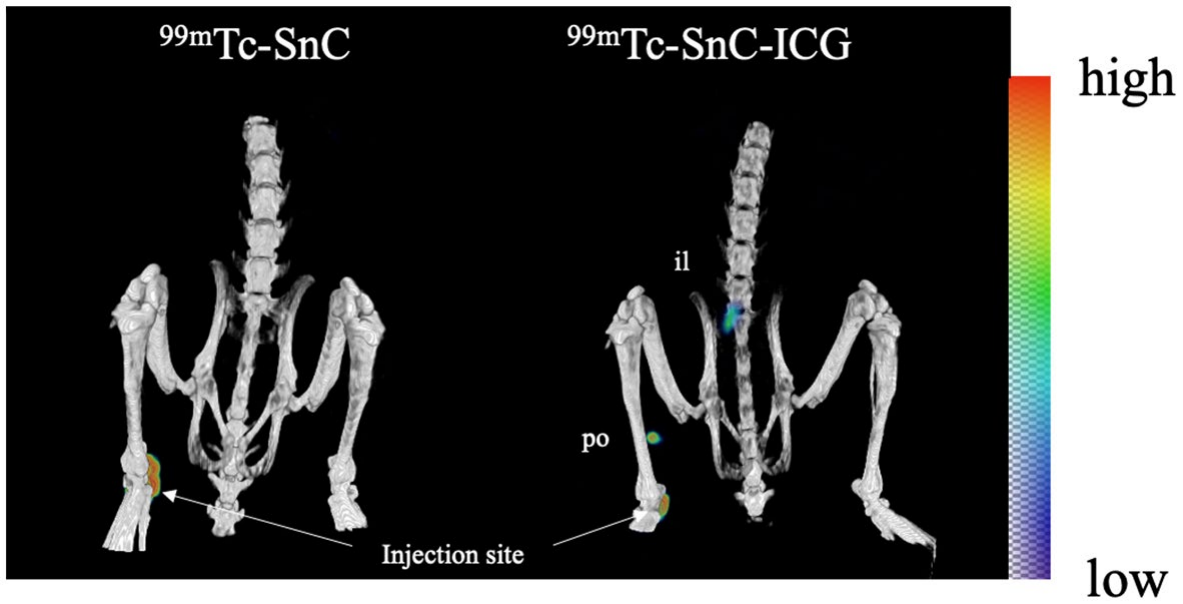
Biodistribution analysis of technetium-99m–stannous colloid (^99m^Tc-SnC) or ^99m^Tc-SnC–indocyanine green mixed tracer (^99m^Tc-SnC–ICG) in rats using single positron emission computed tomography and X-ray computed tomography. Radioactivity of the accumulation site on the images is indicated with a color gradient shown in the rightmost.

Single positron emission computed tomography and X-ray computed tomography (SPECT/CT) visualized hot spots in the tracer injection sites of all rats; however, those within the popliteal and iliac node areas were revealed only in rats administered ^99m^Tc-SnC–ICG (Fig. 4).

**Figure 5:**
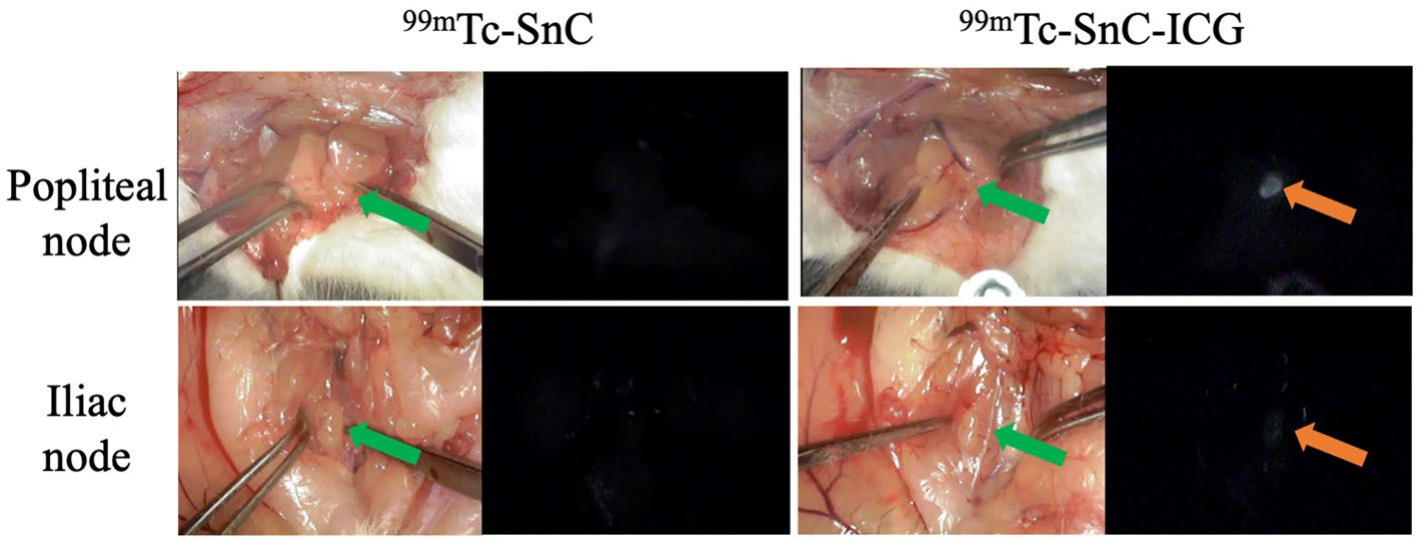
Appearance of regional lymph nodes (LNs) after the administration of technetium-99m–stannous colloid (^99m^Tc-SnC) or ^99m^Tc-SnC–indocyanine green mixed tracer (^99m^Tc-SnC–ICG) using bright-field (left) and near-infrared fluorescence (right) cameras. The arrows indicate regional LNs. Fluorescent nodes are rather ambiguous in this figure because these images were extracted from movies captured by NIR fluorescence camera with a long focal distance for clinical use and high background due to NIR surface-reflections. These nodes were obviously identified with original movies S5 (popliteal node with^99m^Tc-SnC), S6 (iliac node with ^99m^Tc-SnC), S7 (popliteal node with ^99m^Tc-SnC–ICG), and S8 (iliac node with ^99m^Tc-SnC–ICG).

Similarly, the NIR fluorescence camera depicted bright nodes in the same areas only in rats administered ^99m^Tc-SnC–ICG, and the fluorescence intensity appeared stronger in the popliteal versus iliac nodes (Fig. 5).

After the rats were euthanized, the part of the injected foot and the regional LNs (popliteal, iliac, inguinal, caudal, renal, and axillary) were retrieved, and their radioactive counts were measured ex vivo (Table 1). Mean %ID values (adjusted as percentages of injected dose) of each sample are shown in Table 1. The rats administered ^99m^Tc-SnC–ICG had significantly lower (p < 0.001) radioactivity counts in the foot (injection site) and significantly higher radioactivity counts (p = 0.004) in the popliteal LNs than those administered ^99m^Tc-SnC.

**Table 1:** Biodistribution of radioactivity using mixed tracer administration in the rat right foot pad.

%ID	^99m^Tc-SnC	^99m^Tc-SnC–ICG	P
Foot (injection site)	89.22 (10.22)	27.58 (12.27)	0.000
Popliteal	0.07 (0.04)	2.50 (1.16)	0.004
Iliac	0.10 (0.14)	0.69 (0.88)	0.163
Caudal	0.00 (0.00)	0.00 (0.00)	0.930
Renal	0.00 (0.00)	0.01 (0.00)	0.646
Axillary	0.00 (0.00)	0.01 (0.01)	0.108

Each value represents mean (SD) of %ID expressed as the percentage of the injected dose per 10 $\upmu$L of tracer. Statistical significance between technetium-99m–stannous colloid (^99m^Tc-SnC) (n = 4) and ^99m^Tc-SnC–indocyanine green mixed tracer (^99m^Tc-SnC–ICG) (n = 6) was determined using Welch’s t-test.

### Particle size and zeta potential analyses of the tracers

**Figure 6:**
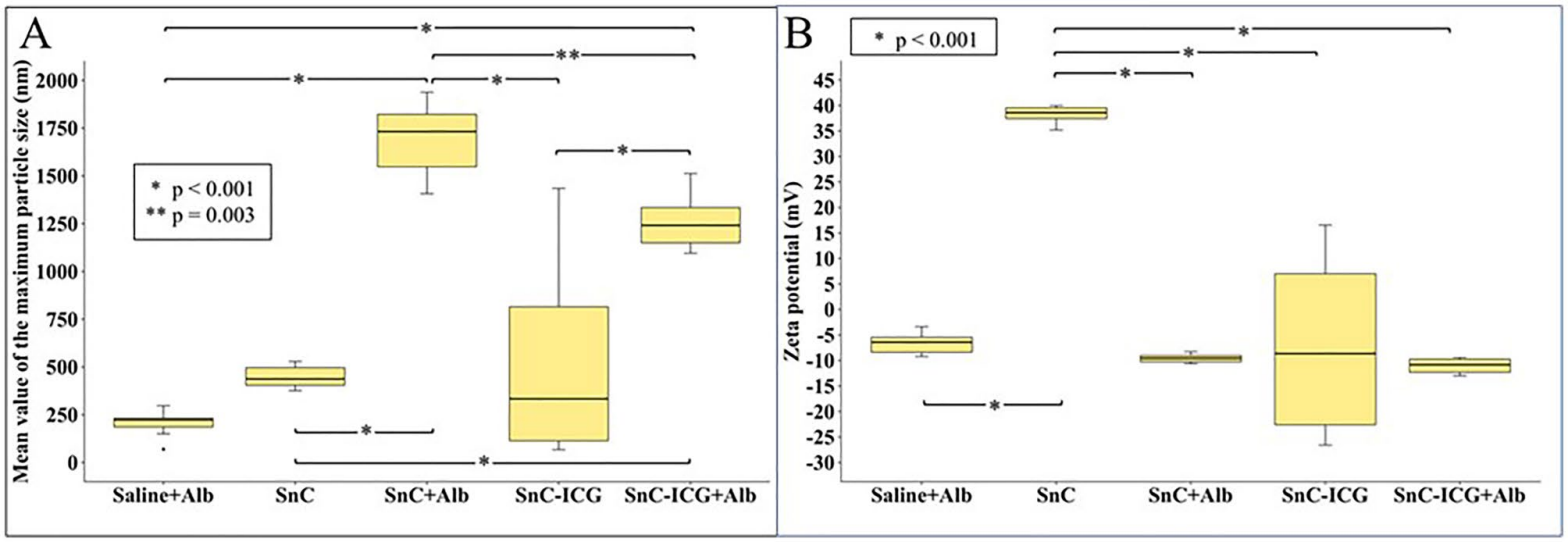
Particle size and zeta potential distributions of the tested samples. A, distribution of mean particle sizes estimated from scattering distribution intensity; B, distribution of peak zeta potentials. Saline + Alb, albumin added saline; SnC, stannous colloid; SnC + Alb, albumin added SnC; SnC–ICG, stannous colloid/ICG mixed tracer; SnC–ICG + Alb, albumin added SnC–ICG.

To investigate the background of the differences in the biodistribution analyses, particle size distributions and zeta potentials of the tracers were examined. Albumin-added samples were also measured to estimate their behaviors in vivo (Fig. 6A). The median particle size of SnC (438 nm) was similar to that previously reported^26^. SnC–ICG administration showed slightly smaller median particle sizes (334 nm) but a much higher size variability (67–1435 nm, range) than SnC (376–529 nm, range). The median particle size of SnC or SnC–ICG increased significantly when albumin was added (1732 and 1241 nm; p < 0.001 and < 0.001, respectively), and the median SnC size was approximately 500 nm larger than the mean SnC–ICG size (p = 0.003). Interestingly, the particle size variability of SnC–ICG was considerably smaller (1095–1513 nm, range) after the addition of albumin, making it comparable with that of albumin-added SnC (1407–1939 nm, range).

The zeta potentials of these samples were estimated as well (Fig. 6B). While SnC showed a significantly higher positive charge than the others (p < 0.001), SnC–ICG showed a wider variety of surface charge that ranged from positive to negative zeta potentials. However, SnC and SnC–ICG showed homogeneous and slightly negative charges after the addition of albumin, resembling the values following albumin-added saline administration.

## Discussion

In this study, we proposed the combination of SnC and ICG as a new tracer (SnC–ICG) for SN navigation, which can be detected with both NIR fluorescence and radioactivity since both are widely used for the same purpose in our country. At 18 h after administration, SnC–ICG showed a better clearance from the administration site and LN retention compared with SnC alone in our rat model. Fluorescence levels of the same LN at the same time point were considerably low but detectable; those with SnC–ICG were significantly higher than those with ICG alone. Two LNs, the popliteal and iliac LN, visualized using an NIR fluorescence camera or SPECT/CT, were thought to be primary LNs from a foot pad in this rat model, since our preliminary study showed two lymphatic routes from a foot pad (Fig. S1 and S2, Movie S9 and S10). These data suggested that SnC–ICG was expected to perform better than SnC alone in the radiocolloid-guided method with an acceptable level of performance in the NIR fluorescence-guided method for SN navigation surgery.

The differences in biodistribution characteristics between SnC–ICG and SnC could be accounted for by the differences in the particle size (Fig. 6A) and surface charge characteristics (Fig. 6B) between the two methods. SnC prepared without albumin exhibited a median particle size of 438 nm, which could be difficult to take up in the lymphatics because only particles less than 100 nm in size are easily taken up by the lymphatics as observed experimentally and clinically^27–30^. SnC–ICG prepared without albumin showed a relatively lower size (334 nm, median) and a much wider size variation (67–1435 nm, range) than SnC and this could have accounted for the better clearance of SnC–ICG from the injection site. SnC–ICG was observed to increase in size (1241 nm, median) and converge to a smaller dispersion (1095–1513 nm, range) after the addition of albumin, which could have contributed to a better retention in primary LNs.

Particle size change in vivo is one of the essential concepts for SN tracers for better clearance from the injection site and longer retention by LNs. Philips et al. showed that a subcutaneous injection of avidin after the administration of biotin-coated liposome greatly enhanced its LN retention in a rabbit model^31^. Similarly, the separate administration of immunogenically modified liposome and pentameric immunoglobulin M in a rat model resulted in their aggregation and retention in LN for prolonged periods of time^32^. Araki et al. also mentioned that small phytate colloidal particles (100–200 nm in diameter) migrate immediately after their injection into LN, where they increase in size upon reacting with ionized calcium and are retained for much longer periods without passing to the downstream nodes^24^. However, they did not investigate the detailed particle profile of phytate colloid mixed with ICG. Our in vivo and in vitro data indicated that SnC and SnC–ICG increase in size in association with serum albumin in vivo.

The difference in surface charge between SnC–ICG and SnC could contribute to the difference in their clearance from the injection site. Tracers with negatively to neutrally charged properties have been shown to be repulsively pushed into the lymphatics by the negatively charged interstitium of the administration site^28,29,33^. Our zeta potential analysis indicated a high positivity for SnC, but a neutral to negative charge for SnC–ICG (Fig. 6B).

The wide variability of SnC–ICG particle sizes and zeta potential distribution suggested an instability of the colloidal product; however, its mechanism requires further elucidation. The zeta potential of SnC was largely positive because its formation occurs via bonding with cationic hydroxide salts (SnOH^+^, Sn_2_(OH)_2_^2+^, etc.), which are formed by the hydrolysis of stannous (II) ions in a solution containing chloride ions^34–36^. Meanwhile, ICG has two negatively charged sulfate residues and a much higher molecular weight than chloride ions; this factor may influence the SnC during the process of nucleation, growth, and cohesion, generating turbulence in the electrostatic repulsion of the colloid particles.

Furthermore, the mechanism of SnC–ICG responsible for its significantly higher fluorescence in primary LNs compared with that of ICG aqueous solution (Figs. 1 and 2) remains unclear. Both tracers in this study showed considerably low levels of fluorescence intensity in the LNs; these data suggest that major portion of ICG are free from SnC in the SnC–ICG mixture. Meanwhile, a histological NIR analysis indicated an intense fluorescence accumulation in the marginal sinus of the regional nodes (Fig. 3), suggesting that some part of the ICG were incorporated in SnC. Our preliminary study with thin-layer chromatography indicated that approximately 55% of ICG was free from SnC in ^99m^Tc-SnC–ICG (Fig. S3). However, it was difficult to explore the actual degree of unity between these two components in ^99m^Tc-SnC–ICG since the particle size of SnC and quantum yield of ICG fluorescence changes with the dilution of the suspension. Nevertheless, the addition of ICG caused considerable changes in SnC in terms of physical conditions and biodistributions, indicating certain interaction between the two tracers.

Our study has some limitations. First, the physical properties of the colloidal samples were measured without the addition of ^99m^Tc. Although the concentration of ^99m^Tc was extremely low, we believe these properties would be altered to some extent in its presence. However, Higashi et al. reported that the colloidal size was the same in the preparations with radiolabeling and those without^37^. Second, we used a normal rat model for the biodistribution analyses of the tracers. However, lymphatic flow of the regional area for tumors is modified in tumor-bearing animals. Third, the pharmacokinetics of SnC–ICG were not investigated because we set a single time point to assess the biodistribution of this tracer with reference to our routine procedure for SN navigation of gastric cancer in a clinical setting. Assessment of different time points would have been more informative but we prioritized the minimization of the number of test subjects for ethical animal welfare reasons. The lymphatic system is much smaller in rats than in humans; thus, the assessment interval would have been too long to estimate the biodistribution in humans. Therefore, the in vivo behaviors of tracers in humans require further verification in clinical settings.

In conclusion, here we proposed a new mixed tracer (SnC and ICG) for SLN detection that can be detected through both radioactivity and near-infrared fluorescence. A biodistribution study in an animal model and physical property analyses indicated that the single administration of SnC–ICG achieved better clearance from the injection site and showed longer retention in primary LNs than the administration of either alone. Although further investigations are required, this new tracer could improve the SLN detection and reduce patient and physician burden in SLN navigation for intra-abdominal and intrathoracic tumors.

## Methods

### Chemicals and reagents

The ICG aqueous solution was prepared using DIAGNOGREEN® purchased form Daiichi-Sankyo Co., Ltd. (Tokyo, Japan). The ^99m^Tc-SnC was dispensed using a SnC Tc-99m kit (Nihon Medi-Physics Co., Ltd., Tokyo, Japan). Isotonic sodium chloride solution (saline) supplied by Otsuka Pharmaceutical Co. Ltd. (Tokyo, Japan) was used as the diluting solution, while Albuminar® 25% purchased from CSL Behring K.K. (Tokyo, Japan) was used to prepare the human serum albumin. The ^99m^Tc was eluted from a 1.1 GBq ^99^Mo-^99m^Tc generator (Ultra-Techne Kow®; PDRadiopharma Inc., Tokyo, Japan). The isoflurane used for general anesthesia in the rats was purchased from Pfizer Japan Inc. (Tokyo, Japan). All reagents were used without further purification.

### Preparation of SnC samples

The concentration of ICG was adjusted to 5 mg/mL using 5 mL of distilled water and then diluted tenfold with saline (final ICG concentration: 0.5 mg/mL). Next, stannous chloride solution, saline, and tenfold diluted ICG aqueous solution at a volume ratio of 5:4:1 (final ICG concentration: 50 $\upmu$g/mL) were gently mixed and incubated at 25 °C for 30 min to prepare the SnC–ICG. As a control, SnC was prepared from stannous chloride solution and saline at a ratio of 1:1 with the same procedure. A few ^99m^Tc-labeled samples (^99m^Tc-SnC–ICG and ^99m^Tc-SnC) were prepared following the same procedure; however, the saline was replaced with the ^99m^Tc extract with approximately 740 MBq/mL radioactivity. For the preparation of the albumin-added samples, 0.15 mL of Albuminar® 25% (final albumin concentration: 12 mg/mL) was added at the final preparation step (final total volume: 3.15 mL each).

### Animals

Ten-week-old female Sprague–Dawley rats (190–230 g) were purchased from Japan SLC, Inc. (Shizuoka, Japan) and housed in plastic cages in an animal housing facility at Chiba University, with a time routine of 12:12 h light/dark cycle, under controlled temperature and humidity, and water and food ad libitum. All animal experiments were performed under general anesthesia. Inhalation of isoflurane was started at a concentration of 4 v/v% and maintained at 2–3 v/v%. All animals were euthanized with an overdose inhalation of isoflurane (5 v/v%) for 20 min before retrieving regional LNs and administration sites. All animal experimental protocols in this study were reviewed (Dou 1–442, Dou 2–458) and approved by the Institutional Animal Care and Use Committee of Animal Experimentation of Chiba University. All methods were carried out in accordance with the relevant guidelines and regulations in Japan. The reporting in the manuscript follows the recommendations in the ARRIVE guidelines (www.arriveguidelines.org).

### NIR fluorescence/radioisotope imaging devices

NIR fluorescence images were obtained by an NIR fluorescence camera (Hyper Eye Medical System®; MNIRC-1000, Mizuho Medical Industry Co., Ltd., Tokyo, Japan). The system enables the real-time speed monitoring of both bright-field and NIR fluorescence video images through a high-contrast charge-coupled device (CCD) camera. Bright-field and NIR fluorescence microscope images of the frozen sections were obtained using a microscope (BX51; Olympus, Tokyo, Japan) equipped with an electron-multiplying CCD camera (iXon-DU897E; Andor Technology Ltd., Belfast, Ireland) and an NIR fluorescence detection filter set (Semrock ICG-A [excitation, 769 nm; emission, 832 nm; dichroic: 813.5–950 nm], Semrock, NY, USA). The in vivo distribution of ^99m^Tc was visualized using a dual-modality imaging instrument with SPECT/CT for small animals (SPECT4/CT, Trifoil Imaging, Chatsworth, CA, USA). The data acquisition was performed at 240 s/projection with a stepwise rotation of 16 projections over 360˚. The SPECT Triumph-RECON software (Trifoil Imaging, Chatsworth, CA, USA) was used to obtain the SPECT images, while the data were reconstructed using a three dimensionally ordered subset expectation maximization algorithm using two subsets and eight iterations.

### Biodistribution analyses of ICG and SnC–ICG using NIR fluorescence camera

With each rat under general anesthesia, 10 $\upmu$L of ICG aqueous solution (50 µg/mL) or SnC–ICG (final ICG concentration: 50 µg/mL) was administered to the right foot pad of four animals each and the injection site was massaged a few times. Eighteen hours later, each rat was laparotomized and regional LNs were observed using an NIR fluorescence camera. Next, all fluorescent LNs were retrieved and embedded in a Tissue-Tek® Optimal Cutting Temperature Compound (O.C.T. compound; Sakura Finetek Japan Co., Ltd., Inc., Tokyo, Japan) and subsequently frozen in liquid nitrogen.

Tissue sections (7 $\upmu$m thick) were prepared and the distribution of fluorescence in the specimen was examined under the NIR fluorescence microscope. Next, the samples were stained with hematoxylin/eosin (HE) and their images were obtained using a digital slide scanner (NanoZoomer S60; C13210-01, Hamamatsu Photonics K.K., Shizuoka, Japan).

The fluorescence intensity of the LN images on the NIR fluorescence camera was estimated using the ImageJ software (NIH, DC, USA). The mean brightness of the fluorescent area determined by a five-time repeated discriminant analysis in the CIE Lab color space on an apparent LN image was used as the representative fluorescence intensity.

### Biodistribution analyses of ^99m^Tc radioactivity

With each rat under general anesthesia, 10 $\upmu$L of ^99m^Tc-SnC–ICG (final ICG concentration: 50 µg/mL) or ^99m^Tc-SnC was administered to the right foot pad of four animals each and the injection site was massaged a few times. The radioactivity of the samples at the time of administration was 120–220 MBq as measured with a Curiemeter (IGC-7; Aloka Co., Ltd., Tokyo, Japan). SPECT/CT imaging studies were conducted 18 h later under general anesthesia. The rats were then sacrificed and the fluorescent nodes were identified using the NIR fluorescence camera. Regional LNs from the injection site and foot were removed to determine their radioactivity counts with an automatic gamma counter (Wizard^2^; Perkin Elmer Japan Co., Ltd., Yokohama, Japan).

### Measurement of particle size and zeta potential

Particle size distribution and zeta potential were measured using a zeta potential/particle size analyzer (ELSZ-1000ZXCK; Otsuka Electronics Co., Ltd., Osaka, Japan) and a data processing software (Photal ELSZ-100 version 5.01; Otsuka Electronics Co., Ltd.). Particle size distribution was estimated based on the dynamic light scattering method of this equipment. The zeta potential was estimated according to the laser Doppler method. Each sample was measured three times at 25 °C.

### Statistical analysis

Mean values of the two groups in the in vivo experiments were compared using Welch’s t-test. Multiple comparisons of mean values from the in vitro experiments were performed using Tukey’s honest significant differences test. All statistical analyses were conducted using the R software version 3.6.3 (The R Foundation for Statistical Computing Platform), and p values < 0.05 were considered statistically significant.

## Competing interests

The authors declare no competing interests.

## Supplementary Information

Supplementary Video 1.

Supplementary Video 2.

Supplementary Video 3.

Supplementary Video 4.

Supplementary Video 5.

Supplementary Video 6.

Supplementary Video 7.

Supplementary Video 8.

Supplementary Video 9.

Supplementary Video 10.

Supplementary Information 1.

## Data Availability

All data generated or analyzed during this study are included in this published article and its additional files.
