# Supplementary material for: Stannous colloid mixed with indocyanine green as a tracer for sentinel lymph node navigation surgery
Source: Sci Rep. 2022 Oct 12;12:17056. doi: 10.1038/s41598-022-21420-z (PMC9556759; doi:10.1038/s41598-022-21420-z)
Supplement: Supplementary file 11 — Supplementary Information 1. [file 41598_2022_21420_MOESM11_ESM.docx]

**Stannous colloid mixed with indocyanine green as a tracer for sentinel lymph node navigation surgery**

Yiting Zhang^1^, Tomoya Uehara^2^, Taro Toyota^3^, Ryusuke Endo^4^, Hisahiro Matsubara^1^,　Hideki Hayashi^1,5,^*

^1^Department of Frontier Surgery, Graduate School of Medicine, Chiba University, Inohana, Chiba-shi chuo-ku, Chiba, Japan. ^2^Department of Molecular Imaging and Radiotherapy, Graduate School of Pharmaceutical Sciences, Chiba University, Inohana, Chiba-shi chuo-ku, Chiba, Japan. ^3^Department of Graduate School of Arts and Sciences, The University of Tokyo, Komaba Meguro-ku, Tokyo, Japan. ^4^Department of Medical System Engineering, Graduate School of Engineering, Chiba University, Yayoi-cho, Chiba-shi inage-ku, Chiba, Japan. ^5^Center for Frontier Medical Engineering, Chiba University, Japan. *Corresponding author, e-mail: [hhayashi@faculty.chiba-u.jp](mailto:hhayashi@faculty.chiba-u.jp)

**Supplementary Information**

**Supplementary Movies S1 - S4:**

**Near-infrared (NIR) fluorescence camera movies for Figure 1**

Movie S1: Popliteal node of the rat administered ICG aqueous solution (ICGaq).

Movie S2: Iliac node of the rat administered ICGaq.

Movie S3: Popliteal node of the rat administered stannous colloid mixed with indocyanine green (SnC-ICG).

Movie S4: Iliac node of the rat administered SnC-ICG.

**Supplementary Movies S5 – S8:**

**Near-infrared (NIR) fluorescence camera movies for Figure 5**

Movie S5: Popliteal node of the rat administered technetium-99m–stannous colloid (^99m^Tc-SnC).

Movie S6: Iliac node of the rat administered ^99m^Tc-SnC.

Movie S7: Popliteal node of the rat administered ^99m^Tc-SnC-indocyanine green mixed tracer (^99m^Tc-SnC-ICG).

Movie S8: Iliac node of the rat administered ^99m^Tc-SnC-ICG.

**Supplementary Figure S1, Figure S2, Movie S9, Movie S10:**

**Analysis of lymphatic routes of rat lower extremity**

Under general anesthesia, the right thigh of a Sprague-Dawley rat (SD; 10 weeks old; female) was shaved, and 0.1 mL of indocyanine green (ICG) aqueous solution (500 μg/mL, x10 concentration of the main experiments) was administered to the right foot pad using a syringe (1 mL used for tuberculin skin test [SS-01T with 27G needle]; NN-2719S; TERUMO, Tokyo, Japan). The injection site was massaged a few times after the administration. One and half hours later, the fluorescent lymphatics were visualized using a near-infrared video camera (MNIRC-1000; Mizuho Medical Industry Co., Ltd., Tokyo, Japan).

A superficial lymphatic route from the right foot pad and a popliteal node were visualized at first. Next, a leg muscle dissection and laparotomy revealed that the superficial route was connected to the iliac node and the popliteal node was connected to the foot pad via another lymphatic route situated in a deeper layer.

Two previous reports indicated that the popliteal lymph node is the primary node and the iliac node is the secondary node for mice and rats^S1-4^. In contrast, our preliminary experiments showed two lymphatic routes from the foot pad: the superficial route to the iliac node and the deeper layer to the popliteal node (Figure S1, S2, Movies S9, S10). The SD rats used in this study apparently had two routes that divide into the popliteal and iliac lymph nodes from the foot pad, although this finding differed from those in previous reports.


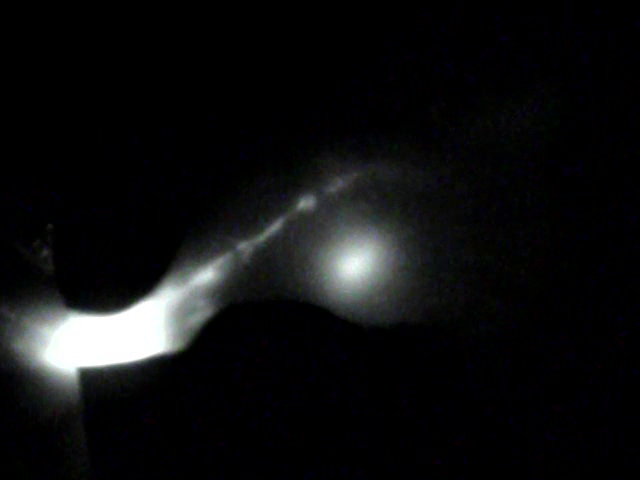

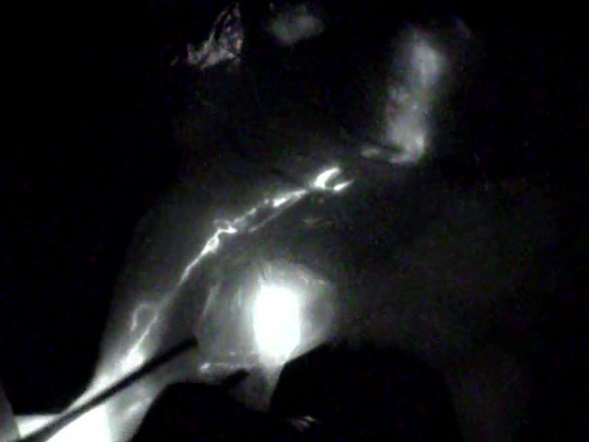


Superficial route

Deeper route

Popliteal node

Iliac node

Popliteal node

Injection site

Figure S1 (left) and S2 (right): Two lymphatic routes were revealed. Superficial route connected to iliac node and deeper one connected to popliteal node.

Movie S9: Original movie from which Figure S1 was obtained.

Movie S10: Original movie from which Figure S2 was obtained.

-------------------------------------------------------------------------------------------------------

**Supplementary Figure 3:**

**Thin-layer chromatography analysis of ^99m^Tc-SnC, ^99m^Tc-SnC-ICG and ICG**

Thin-layer chromatography (TLC) was performed based on the techniques specified in the Interview Form (IF) of ^99m^Tc-stannous colloid kit for Japan Hospital Pharmacist Association described by Nihon Medi-Physics Co., Ltd^S5^. In this analysis, silica gel plates with an aluminum back layer support (105554, Merck KGaA, Darmstadt, Germany) were used, and all samples were developed with methanol as the mobile phase solvent since ICG is insoluble in ethyl methyl ketone. The distribution of ^99m^Tc on the plates was measured with a radiochromatogram scanner (MiniGITA, Elysia Raytest, Straubenhardt, Germany). Chromatograms were recorded and analyzed using the MiniGITA software (Elysia Raytest, Straubenhardt, Germany). The fluorescent spot distributions were visualized using near-infrared fluorescent camera (MNIRC-1000; Mizuho Medical Industry Co., Ltd., Tokyo, Japan). The transfer distance of fluorescent spots was analyzed by “plot profile” function of image analysis software ImageJ (NIH, USA).

Radioactivity scan of the ^99m^Tc labeled samples indicated that 8.4% of ^99m^Tc was free from ^99m^Tc-SnC-ICG. NIR fluorescence scan indicated that 55% of the ICG was free from ^99m^Tc-SnC-ICG but the rest was missing.


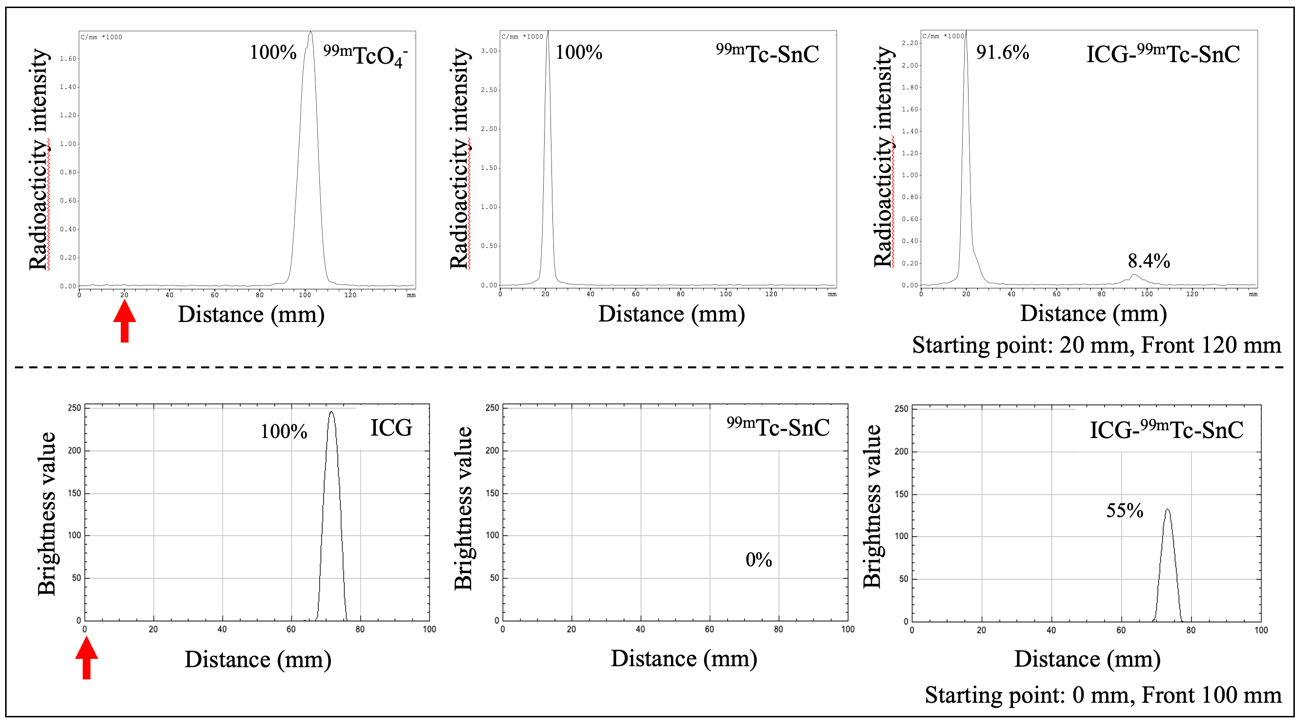
 **Figure S3 Thin-layer chromatography analysis of ^99m^Tc-SnC, ^99m^Tc-SnC-ICG and ICG** (A) ^99m^TcO_4_^-^, ^99m^Tc-SnC, and ^99m^Tc-SnC-ICG were developed, and distribution of radioactivity was scanned. (B) ICG aqueous solution, ^99m^Tc-SnC, and ^99m^Tc-SnC-ICG were developed and distribution of NIR fluorescence was scanned. Red arrows indicate the starting point.

^99m^Tc-SnC-ICG

^99m^Tc-SnC-ICG

**References for supplemental information**

S1 Tilney, N. L. Patterns of lymphatic drainage in the adult laboratory rat. *J. Anat.* **109**, 369-383 (1971).

S2 Suami, H., Chang, D. W., Matsumoto, K. & Kimata, Y. Demonstrating the lymphatic system in rats with microinjection. *Anat Rec (Hoboken)* **294**, 1566-1573, doi:10.1002/ar.21446 (2011).

S3 Lee, S. H. *et al.* Primo vascular system in the lymph vessel from the inguinal to the axillary nodes. *Evid. Based Complement. Alternat. Med.* **2013**, 472704, doi:10.1155/2013/472704 (2013).

S4 Suami, H. & Scaglioni, M. F. Lymphatic Territories (Lymphosomes) in the Rat: An Anatomical Study for Future Lymphatic Research. *Plast. Reconstr. Surg.* **140**, 945-951, doi:10.1097/PRS.0000000000003776 (2017).

S5 Medi-Physics, N. *Tin Colloid Tc-99m Kit interview form*, <<https://www.nmp.co.jp/sites/default/files/2018-09/IF_Sn_Sn-kit.pdf>> (2017).
